# Nanoantibiotics: Functions and Properties at the Nanoscale to Combat Antibiotic Resistance

**DOI:** 10.3389/fchem.2021.687660

**Published:** 2021-05-13

**Authors:** M. Mustafa Mamun, Adeola Julian Sorinolu, Mariya Munir, Eric P. Vejerano

**Affiliations:** ^1^ Center for Environmental Nanoscience and Risk, Department of Environmental Health Sciences, University of South Carolina, Columbia, SC, United States; ^2^ Civil and Environmental Engineering, The William States Lee College of Engineering, University of North Carolina, Charlotte, NC, United States

**Keywords:** nanoparticle, drug delivery, MRSA, nanomaterial, penicillin, vancomycin, Trojan horse

## Abstract

One primary mechanism for bacteria developing resistance is frequent exposure to antibiotics. Nanoantibiotics (nAbts) is one of the strategies being explored to counteract the surge of antibiotic resistant bacteria. nAbts are antibiotic molecules encapsulated with engineered nanoparticles (NPs) or artificially synthesized pure antibiotics with a size range of ≤100 nm in at least one dimension. NPs may restore drug efficacy because of their nanoscale functionalities. As carriers and delivery agents, nAbts can reach target sites inside a bacterium by crossing the cell membrane, interfering with cellular components, and damaging metabolic machinery. Nanoscale systems deliver antibiotics at enormous particle number concentrations. The unique size-, shape-, and composition-related properties of nAbts pose multiple simultaneous assaults on bacteria. Resistance of bacteria toward diverse nanoscale conjugates is considerably slower because NPs generate non-biological adverse effects. NPs physically break down bacteria and interfere with critical molecules used in bacterial processes. Genetic mutations from abiotic assault exerted by nAbts are less probable. This paper discusses how to exploit the fundamental physical and chemical properties of NPs to restore the efficacy of conventional antibiotics. We first described the concept of nAbts and explained their importance. We then summarized the critical physicochemical properties of nAbts that can be utilized in manufacturing and designing various nAbts types. nAbts epitomize a potential Trojan horse strategy to circumvent antibiotic resistance mechanisms. The availability of diverse types and multiple targets of nAbts is increasing due to advances in nanotechnology. Studying nanoscale functions and properties may provide an understanding in preventing future outbreaks caused by antibiotic resistance and in developing successful nAbts.

## Introduction

The global emergence and spread of antibiotic resistance are preeminent public health issues of the 21st century with overwhelming healthcare costs and increased morbidity and mortality. The rising antibacterial resistance cases pose a continual threat when the number of new antibiotics is grossly limited (Nikaido, 2009; Laxminarayan et al., 2016). Furthermore, the shortage of novel molecules has amplified the resistance problem requiring alternative approaches such as nanoantibiotics (nAbts) to emerge. The shrinking antibiotic pipeline has shifted the research paradigm (Doron and Davidson 2011; Barlam et al., 2016) of our understanding of how bacteria function in the presence of an antibiotic (Laws et al., 2019) and or nanoparticles (NPs) (Pelgrift and Friedman 2013), leading to the development of new effective strategies to reconfigure the existing antimicrobial molecules. Antibiotics functionalized with NPs (NPs) or engineered pure antibiotics in the nanoscale have the intrinsic capacity to penetrate bacterial cell membrane barriers and reach specific sites with a higher level of accuracy and stability than free antibiotic molecules (Jijie et al., 2017). Recently, engineered NPs have exhibited target-specific delivery potentials (Singh and Lillard 2009; Capeletti et al., 2019; Ruehle et al., 2017; Zhao et al., 2010) and improved binding affinities (Tassa et al., 2010; Wigginton et al., 2010; Schwaminger et al., 2017; Kim et al., 2018) inside the localized compartments of bacteria. The interaction of nanoscale antibiotics with bacterial intracellular components is central to applications such as antibiotic delivery, drug carriers, cell membrane penetration to reach target sites, and protein synthesis disruption. Therefore, nAbts are considered promising alternative strategies to overcome antibacterial resistance and treatments in clinical infections. Research and applications of nAbts have grown significantly because resistant bacteria render most antibiotics ineffective in their molecular forms. Bacteria have evolved multiple mechanisms to tackle these antibiotics, such as degradation by intracellular β-lactamase enzymes, drug permeation changes across membranes, and alteration of proteins that bind with antibiotic targets (Blair et al., 2015).

Novel antibiotics are the derivatives of clinically successful old antibiotics already in use. Most new β-lactams target the prime resistance mechanism—the β-lactamase enzyme destroys the β-lactam rings of the antibiotics. Potentially, most β-lactam and non-β-lactam antibiotics in their free molecular forms are either ineffective or less functional against resistant microbes. Bacterial efflux pumps eject, and antibiotic-inactivating enzymes deactivate the drug molecules before reaching intracellular target sites (Pang et al., 2019). A drug delivered *via* or in nanoparticulate form of the same concentration has much more inhibitory effects on bacterial growth because nanoscale drug carrier systems can deliver and bind with intracellular targets, thus inhibiting bacterial growth and metabolism and ultimately leading to death (Jijie et al., 2017). The smaller size of NPs compared to their bulk chemical analog alters their physicochemical properties resulting in an enhanced effectivity. The size of NPs can change the pharmacokinetics, specifically translocation, distribution, absorption, elimination, and metabolism of the conjugate’s nanoformulation. These changes enable NPs to surpass the bacteria’s multilayered barriers and be delivered to target sites. To confront antibiotic resistance, we need an arsenal of tools in the nano-regime to reengineer the existing antibiotic molecules with NPs. By themselves, nanostructured particles/materials can enable many antibacterial strategies in the absence of an antibiotic molecule (Hopf et al., 2019; Wang et al., 2020b), which we excluded in this review. This article will inform us of the molecular mechanisms and functional features of nAbts. We will focus this review on the mechanistic insights of nAbts. This minireview deals with the following research questions:

Q1. What are nAbts, and why are they important?
Q2. What are the physicochemical characteristics and antibacterial mechanisms of nAbts that trigger biological hazards inside bacteria?
Q3. What are the common types and functions of nAbts to restore antibiotic efficacy?

## nAbts

### Definition and Concept

nAbts is one of the promising applications of nanotechnology (“Nano on Reflection” 2016) that employ physicochemical conjugation of tiny particles with antibiotics (Soares et al., 2018) or artificially synthesized pure antibiotic molecules in size range of ≤100 nm in at least one dimension. This new Frontier of antimicrobials revitalizes the existing arsenal of drugs by making them effective against a range of clinically meaningful bacteria with the help of nanoscale antibiotics reengineering. Depending on the mode of action, structures, and clinical applications of nAbts, drug molecules’ design and placement can occur at the nanoconjugate’s core or corona. However, the molecules can be physically or chemically attached to NPs without any core-corona structures. These antibacterial molecules linked with NPs, which are either chemically pure by nature (e.g., Si, Fe, Au, Ag, Ti, etc.) consisting of elemental properties or pure NPs with their surface-functionalized (coatings) with different chemical moieties such as carboxylate (-COOH), citrate, PVP, various polymers, etc.

### Types and Examples

NPs are as small as 1 nm to as large as 100 nm to fit into the nanoscale regime for various transport, drug delivery, and controlled drug release purposes in bacteria and humans. Characteristically, these NPs are natural, incidental, or engineered, but most applications belong to the engineered category. In microbiological applications, NPs usually consist of inorganic elements and transition metals (Ag, Au, Pt, Zn, Ti, Al, Fe, Ni, Cu, Si, quantum dots, etc.), their oxides (ZnO, TiO_2_, Fe_3_O_4_, CuO, SiO_2_, etc.), and various carbon-based organic structures [liposomes, micelles, dendrimers, fullerenes, carbon nanotubes (CNTs), and graphene and its derivatives]. A detailed list of available NP types, their synergy with antibiotics, and the associated functions are given in Tables 1, 2, in *Nanoparticle-Functionalized Antibiotics* section. The linkage of antibiotics with engineered NPs is crucial because the type of surface charge (positive, negative, neutral, or zwitterionic) and their densities determine the effectiveness of bacterial killing. Negatively charged particles functionalized with antibiotics have been experimented with mainly to increase the antimicrobial potentials of nanoconjugates (Miller et al., 2015). Functionalizing the outer surface of NPs with charged compounds can significantly enhance the combined effectiveness of nAbts conjugate that kills both Gram-positive and Gram-negative organisms such as *Escherichia. coli*, *Staphylococcus aureus*, Methicillin-resistant *Staphylococcus aureus* (MRSA) (Wang et al., 2014), etc.

**TABLE 1:** Different types of membrane-bound nAbts and their associated functions.

Membrane-bound NPs	Size/shape	Conjugated antibiotic	Conjugate’s chemistry	Targeted bacteria	Target site	Mechanism of action	Reference
Liposome	86.23 ± 14.02 to 109.33 ± 9.66 nm, spherical vesicle	Vancomycin (Van)	Polyanionic DNA nanostructured gels encapsulated with vancomycin and encaged within a cationic liposomal vesicle (Van_DNL) is formulated by non-covalent aromatic stacking/hydrogen bonding/electrostatic/hydrophobic interactions.	*Staphylococcus aureus* (ATCC BAA-1721)	Cell membrane	As a backbone building block, DNA nanostructured liposomes’ self-assembly significantly enhances Van’s loading capacity. High uptake efficiency of Van-DNL *via* intracellular delivery in bacteria enables sustained temporal release of vancomycin and constant ROS generation.	Obuobi et al. (2020)
Micelle	77 nm, spherical	Vancomycin (Van), Ciprofloxacin (CIP)	A pH- cleavable Hydrazone bond connects the carboxyl group of targeting ligand Van with an acetyl group of poly (ε-caprolactone) PEL initiated by a poly (ethylene glycol) (PEG)	*Escherichia coli TOP10, Pseudomonas aeruginosa* PA01	Cell wall and cell membrane	Van and CIP-loaded micelles act as stimuli-responsive nanocarriers inside the host cell infection site. Target specific adhesion process interacts the drug conjugates with bacterial lipid membranes and injects on-demand sustained drug release profusely.	Chen et al. (2018)
Dendrimer	52.21 ± 0.22 nm, spherical	Vancomycin (Van)	Van and dendrimer molecules are encapsulated at the core of lipid-dendrimer hybrid NPs (LDHNs) and coated with a lipid shell. The LDHNs have a high negative charge, monodisperse, and did not form aggregation. The dendrimer’s Multivalent binding capacity can entrap very high Van at the core by hydrogen bonding.	*Staphylococcus aureus* (ATCC25922), Methicillin-resistant *Staphylococcus aureus* (MRSA) (ATCC BAA-1683)	Cell membrane	High entrapment efficiency and Van’s loading capacity inside the smaller-sized LDHNs lipid carriers ensure successful penetration and sustained drug delivery inside the target site's cell membrane.	Sonawane et al. (2016)

**TABLE 2:** Different types of metallic nAbts and their associated functions.

Metallic NPs	Size/shape	Conjugated antibiotic	Conjugate’s chemistry	Targeted bacteria	Target site	Mechanism of action	Reference
Silver (Ag)	4 nm, spherical	Ampicillin (Amp)	Citrate coated AgNPs can load 1.06 × 10^-6^ mol of Amp by functionalization on to AgNPs surface	*Pseudomonas aeruginosa*, *Vibrio cholerae*, *Escherichia coli*	Cell wall and cell membrane	Approx. Five hundred twenty-three molecules of Amp/AgNP bring a combined antibacterial effect of Amp and AgNP, resulted in a ten-fold decreased amount of Amp-AgNP conjugate to kill β-lactam resistant bacteria at a faster rate compared to free Amp.	Brown et al. (2012)
Gold (Au)	10–12 nm, spherical	Gentamicin	The three NH2 groups of gentamicin non-covalently strongly bind with AuNP by hydrogen bonding.	*Staphylococcus aureus* (ATCC29213)	Cell wall	AuNP conjugates selectively bind with the cell wall, penetrate and deliver a large gentamicin amount.	Ahangari et al. (2013)
Gold (Au)	4–5 nm, spherical	Vancomycin (Van)	Phenyl group of Van attaches with AuNP by Au-S bonds. Each Au NP links with approx. 31 Van molecules on its surface.	Vancomycin-resistant enterococci, *Escherichia coli*	Cell membrane	Van-capped AuNP serves as a multi/polyvalent inhibitor to the bacterial cell membrane.	Gu et al. (2003)

### The Trojan Horse Strategy

In the biotic world, most cells/organelles and pathogens have specific gateways that prevent the entry of exogenous materials that are not vital to their processes. Materials that can be incorporated with or rendered as NPs can efficiently traverse cellular membranes because of their small size (Prabha et al., 2016). NPs of different sizes, shapes, and surface properties can be artificially engineered. Alternatively, NPs containing similar chemical composition to cells and their components such as lipids or nucleic acids can bypass these gateways. The introduction of nAbts triggers the Trojan horse phenomenon once they arrive at the cellular or bacterial gateways. The first part of this section discusses the theoretical concepts, while the latter part provides examples.

The successful entry of a sufficient concentration and enhanced efficacy of drugs in cells can occur due to these processes and factors. First, nanoscale conjugates can hide antibiotic molecules during cellular entry. Second, bacterial cells are less likely to expel antibiotics through the efflux pumps if delivered at an enormous particle number concentration. Third, selective delivery of antibiotics at a sustained release rate to the infection site is enhanced when in the nanoscale form. Lower doses of nano-enabled multiple antibiotics are more effective than their molecular form due to differential drug release kinetics. Fourth, some specific polymeric NPs have an enhanced biodegradability in infected cells. Once introduced, polymeric NPs can cross multiple membranes, degrade inside the diseased cell, and selectively bind at the infected components such as cytoplasm or macrophage or phagolysosome. Fifth, NPs preferentially accumulate inside cells. NPs can trap microbes to decrease the burden of infections and block extracellular bacterial entry into macrophages. Sixth, NP’s stimuli-response features, e.g., temperature, pH, light, ultrasound, magnetism, oxygen or carbon dioxide levels, ionic strength, etc., can be modulated. Seventh, NPs can activate multiple simultaneous antimicrobial mechanisms. It is improbable for a microbe to mutate. Therefore, bacteria are less susceptible to develop resistance if exposed to nAbts. In sum, once these nanosystems cross the initial barrier of the cell membrane, the Trojan horse effect is activated by releasing antibiotic molecules to the infection site or interfering with the metabolic machinery of bacteria.

For instance, lipid-based micellar or liposomal NPs can mimic bacterial cell surfaces (Gao et al., 2013) that have stimuli-responsive features and facilitate uninterrupted cellular entry *via* membrane adhesion and fusion (Wang et al., 2020). Methicillin-resistant *Staphylococcus aureus* (MRSA), a drug-resistant strain of *S. aureus*, has been successfully eradicated in the laboratory using amphiphilic liposomes with a high drug loading capacity (Gao et al., 2021). A gold NP can accommodate 31 vancomycin molecules and are potentially delivered at the infection site with precision (Gu et al., 2003). This strategy is mainly used for multidrug-resistant Gram-positive and Gram-negative bacteria. A biodegradable polymer, i.e., poly (lactic-co-glycolic acid) (PLGA) NP, can be transported through multiple membranes *via* phagosomal/endosomal membrane, then into the cytoplasm, and finally, to the affected site (Kalluru et al., 2013). However, one study (Faria et al., 2012) found that PLGA coupled with isoniazid is directly transported to *Mycobacterium* BCG lysosomes in the macrophages. For acute and persistent infections, diseased cells selectively treated by nAbts are more efficient than their molecular form even when only a fraction of the drug was released from the PLGA NP conjugates (Toti et al., 2011). Graphene-oxide (GO) has a unique degree of *in vivo* specificity, biodegradability, low cellular toxicity, and enormous drug loading capacity (McCallion et al., 2016). When administered *in vivo*, GO preferentially accumulates in lung cells (De Maio et al., 2020) and inhibits the growth of *Mycobacterium tuberculosis* (Mtb), which is the prime microbial agent of *tuberculosis* infection (Maio et al., 2019).

### Functional Features

Generally, with decreasing size of NPs, the specific surface area of the conjugate’s colloidal system increases, thus resulting in more antibiotic molecules to be functionalized onto NP’s surface. One of the main challenges of antibiotic delivery in bacteria is that drug efficacy significantly diminishes before entering the target site because of primarily being excreted by the efflux pumps (Li et al., 2015). The drug carrier should also possess relatively low toxicity and inert for clinical use while successfully disrupting the bacterial metabolic process. Inorganic NPs such as silica with porous structures are essential for microbiological applications (Vallet-Regí et al., 2007).

Nanomaterials specially designed with porous features have pivotal drug delivery and functionalization applications. For instance, specific amorphous or crystalline structures, mesoporous materials with cylindrical or cage-type void spaces, contain exceptionally high internal surface areas (Li et al., 2016). Easy surface-functionalization, tunability of the particle size and nature of the surface, tunable pore shape and volume, and controllable drug delivery systems from these mesoporous materials have gained a burgeoning interest. Mesoporous structures mainly composed of silica, alumina, titania, carbon, and metal oxides with pore sizes between 2–50 nm facilitate higher adsorption of antibiotic molecules. The key advantages come from their biocompatibility, low toxicity, simple functionalization, and encapsulation strategies with organic molecules. Microporous inorganic structures with an extremely narrow pore size range of 0.5–2 nm (Dou et al., 2021) serve as an excellent platform for adsorption and ion-exchange processes inside cells. The uptake process triggers the interactions of microporous and mesoporous NPs with bacteria. The high surface area (>500 m^2^/g) of these porous NPs allow significant uptake of these substances in cellular components and penetration through bacterial lipid bilayers based on surface properties (i.e., hydrophilicity, hydrophobicity, or functionality) (Wang et al., 2019). Large specific surface area, pore volume, and pore size can increase the adsorption capacity of drugs and therefore encapsulate a large concentration of antibiotics that perform their controlled release at the bacterial target site.

Scientists believe that NPs are more toxic than their larger counterparts of the same chemical composition (Luyts et al., 2012) that target cellular and intracellular elements, ultimately killing bacteria. Multiple lines of evidence suggest that pure NPs (without any antibiotic or antimicrobial molecules) are lethal to bacteria and can substitute as an alternative to antibiotics (Singh et al., 2020; Lee et al., 2019; Raghunath and Perumal, 2017). This concept has gained increasing attention for treating bacterial infections instead of clinical antibiotics (Zhao and Jiang, 2013), such as penicillin or vancomycin. However, debates exist regarding the toxicity of pure NPs raising questions on their practical clinical applications even though pure NPs exhibit little to slower rates of antimicrobial resistance (Brown et al., 2012; Su et al., 2019). In this review, we discuss the tunable features of nAbts, excluding any pure nanoparticle-based applications that can restore antibiotic efficacy.

## Why nAbts?

### Rationale Behind nAbts

Antibiotics generally kill bacteria by disrupting the proton motive force across the cell membrane, diminishing the bacteria’s ability to store or generate energy, inhibiting protein synthesis, and breaking the cell wall’s structural components, etc. (Kohanski et al., 2010). Scientists investigate “antibiotic-like” molecules and structural features that destroy bacteria in a completely unknown and new mechanism to microbes. Most conventional antibiotics require multiple doses in a systematic release, whereas nAbts bring added benefits of a target-specific, controllable sustained release that can be administered in a single dose. NPs have multifunctional “intelligent” antimicrobials that behave as stimuli-responsive to interact with bacterial cell wall/membrane surfaces, leading to enhanced penetration across membranes and drug delivery at target sites. Introduction of nano-enabled functions is diversifying the arsenal of antibiotics and repurposed drug molecules that target bacterial sites and adjust specific features on-demand. Antibiotics engineered at the nanoscale have exciting roles in defining antibacterial activity and site-specific selectivity.

### Properties

**FIGURE 1:**
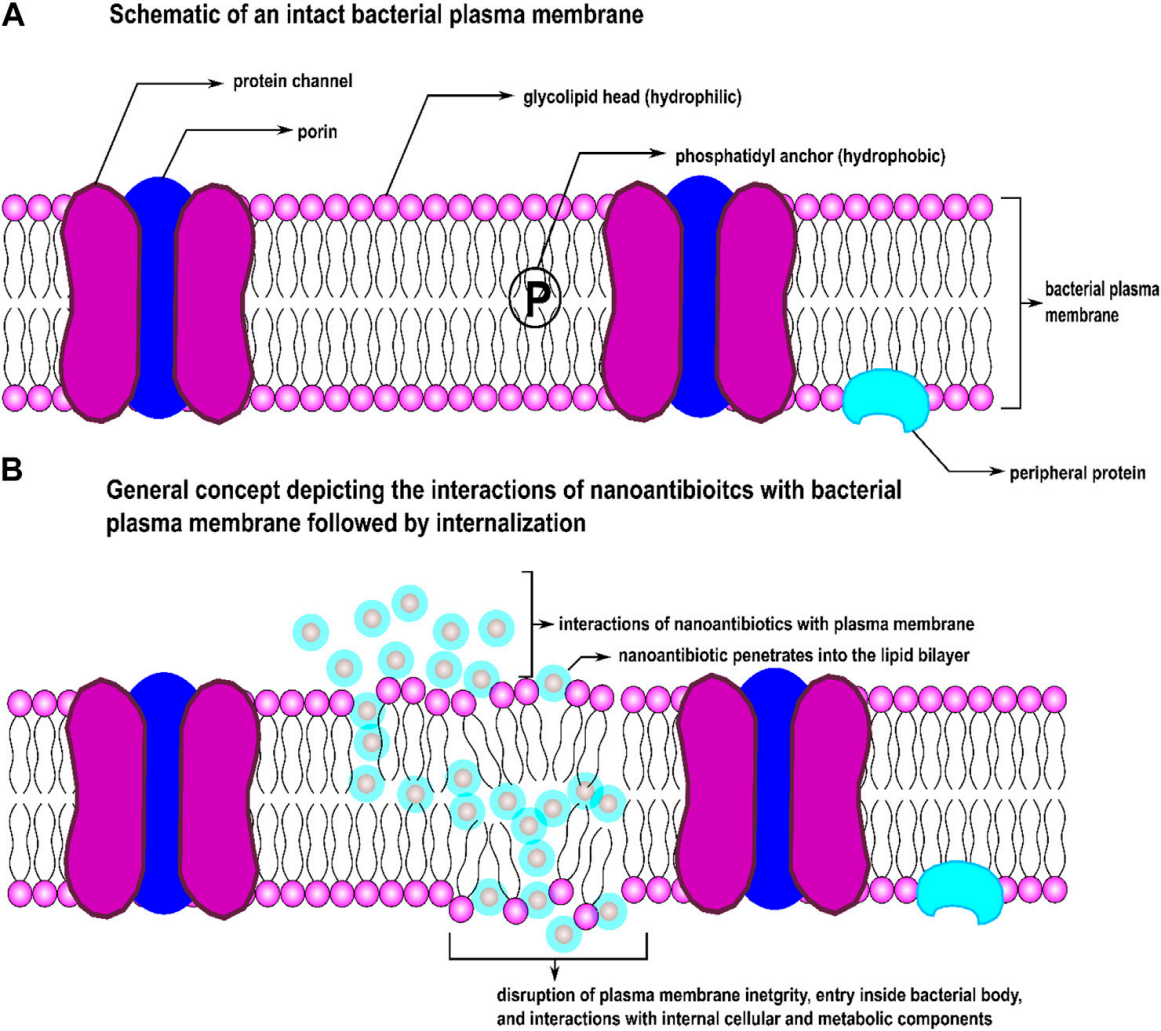
Schematic of **(A)** an intact bacterial cell membrane and **(B)** effect of nAbts on the integrity of a bacterial cell membrane.

Functionalization of a drug at nanoscale system particles, a drug’s destruction mode can be modified that efficiently destroy resistant strains (Miller et al., 2015;Wang et al., 2014; Brown et al., 2012) by suppressing or overcoming bacterial drug resistance. In comparison with the molecular materials, NPs have a high surface-to-volume ratio and specific surface area; therefore, such nanoscale particles possess exceptionally high contact area than molecular materials of the same mass. The morphological properties of NPs can be independently controlled. When particles become smaller for some metal oxide NPs, their lattice constants expand because the electrostatic force decreases with increasing charge densities and metal ions concentration. This feature results in a surge in oxygen vacancies in the crystal structures. This size-dependent expansion and contraction of lattice parameters create different equilibrium spacing between atoms inside an NP compared to their bulk single crystals, resulting in higher adequate surface tension on the surface of NPs. At the nanoscale, these modifications come from NP’s unprecedented shift in physicochemical properties that categorize into three distinct mechanisms: oxidative, metal-ion release, and non-oxidative. Organic NPs (ONPs) synthesized from organic compounds such as proteins, lipids, carbohydrates, nucleic acids, etc., can directly interact with bioactive components of bacteria. Some metallic NPs and ONPs can simultaneously exert these three distinct antimicrobial mechanisms when functionalized with antibiotics (Zaidi et al., 2017). Oxidative mechanisms include: stress induction *via* reactive oxygen species (ROS) and free-radical generation, inhibition of electron transport chain, plasmid damage, disruption of the cell wall, DNA damage, disruption of enzyme activity, metal-ion release include the disintegration of metal ions from nanoconjugates into dissolve form, plasmid damage, DNA damage, interruption of electron transport chain, disruption of enzyme activity, and non-oxidative characteristics such as surface energy, size shape, surface roughness, types and materials of nanoconjugates, cytoplasm release, atomically thin structures, zeta potential (surface charge), stability, increased specific surface area/volume ratio, high surface reactivity, poly- or multi-valency (Fasting et al., 2012), magnetism, conductivity, bioavailability of NPs, drug delivery systems at target sites, physical interactions between particles and cell wall/membranes, crystal phase properties and structures, and particle-antibiotic interface, etc. These features allow proper contact with bacterial cells and enhanced penetration capacities crossing the cell membrane while actively interfering with cellular elements and metabolic machinery. Figure 1 depicts a simple schematic of how nanoscale particles function as carriers of drug molecules across bacterial membrane barriers. Various NPs have been stabilized and functionalized with different antibiotic molecule classes to overcome antibiotic resistance (Jijie et al., 2017; Baptista et al., 2018).

### Antibacterial Mechanisms

Organisms causing intractable infections evolve antimicrobial resistance against novel antibiotic or combination antibiotics primarily because once a species gains tolerance to an antibiotic, it ultimately overcomes combinatory drug effects yielding multidrug-resistant bacteria. This scenario occasionally spurs the resistance development by promoting bacterial production that was not destroyed instantly (Liu et al., 2020). The main pathway to this single or multiple drug resistance is antibiotic tolerance by promoting or suppressing, or transmitting resistance (Fridman et al., 2014; Levin-Reisman et al., 2017; Liu et al., 2020; Berti and Hirsch, 2020). However, we believe that bacterial resistance in response to NPs is considerably slower and hard to develop due to the diverse types of NPs that possess their unique antimicrobial mechanism and/or bind with multiple targets. Bacteria would require various simultaneous action mechanisms from multiple simultaneous gene mutations in the same bacterial cell (Lee et al., 2019; Zhao and Jiang, 2013; Fischbach, 2011) to counteract NPs. When antibiotics are conjugated with NPs, nanoscale systems deliver antibiotics at the target sites and simultaneously pose various antibacterial mechanisms (Zaidi et al., 2017). Therefore, NPs have added functionalities compared to the effects of one drug or multiple drugs in combination when treating drug-resistant bacteria that produce multiple antibacterial mechanisms. Multidrug-resistant microbes possess the most extraordinary challenge toward all kinds of antibiotics. Synergistic antibiotic release from NPs or multiple antibiotics loaded on NPs bring added advantages in the effective and faster killing of bacteria. The increased cytotoxicity of the nanoparticle-based drug synergy compared to the free drug conjugates is derived from the various drug release kinetics and predefined stoichiometric ratio present in the same drug-delivery nanocarrier (Aryal et al., 2010).

### Release Into the Environment

Both incidental and accidental antibiotic release can be degraded in the environment, leading to resistance development. With the given diverse usage of antibacterial drugs, it is virtually impractical to limit the exposure of antibiotics discharged into the environment. In clinical settings, the application of antibiotics only focuses on the delivery and efficacy inside biological cells. Ideally, drug molecules should rapidly deactivate upon release into the environment, and this desirable feature is missing in most antibiotics. The discovery of bacterial responses to external stimuli (Hu et al., 2018; Kimkes and Heinemann, 2020) suggests that nanostructured antibiotics can provide a generic platform for synthesizing “smart” antibiotics by modifying the assembly or disassembly of nanoscale dynamic properties. Zheng and co-authors have designed such “smart” nAbts that can rapidly disable and thus reduce antibiotics footprints in the environment by enabling this “plug-to-activate” and “plug-to-deactivate” concept of environmentally benign nAbts (Zheng et al., 2020).

### Comparison Between Molecular Antibiotics and nAbts Using β-lactam as a Model

Below is a comparison of mechanisms on how molecular and nano-based antibiotics kill bacteria. β-lactam antibiotics kill bacteria by cell lysis *via* multiple mechanisms: (i) inactivating the transpeptidase enzyme that involves in bacterial peptidoglycan cell wall cross-linkages, (ii) inhibiting the penicillin-binding proteins (PBPs), the enzymes responsible for peptidoglycan biosynthesis of the bacterial cell wall, and (iii) degrading Ftsl (PBP3), an essential protein complex essential for bacterial cell division machinery (Chung et al., 2009), causing cell death. For β-lactam-β-lactamase inhibitors, the β-lactamase inhibitors (BLIs) inactivates BLs, so the accompanying β-lactam can bind with target PBP, ultimately rupturing the cell wall. ROS generation by nAbts can cause DNA damage, protein denaturation, membrane impairment, and compromise membrane integrity (Brayner et al., 2006; Zhang and Rock, 2008). The NPs associated with antibiotics can cause biomechanical damage by dissolving and releasing metal-ions (Nel et al., 2009) to disrupt cellular structures physically. This process inhibits bacterial electron transport chains. Additionally, signal transduction impedes ATPase activity and ribosome subunits to bind with tRNA altering metabolic processes such as protein regulation, fat, carbohydrate, and energy metabolism, ultimately suppression of bacterial growth and cell death (Zhao et al., 2010; Cui et al., 2012; Lemire et al., 2013; Tarrat et al., 2015). The surface and morphological properties of NPs, such as various sizes and shapes can affect bacterial cellular uptake and delivery of therapeutic agents (Ohta et al., 2016).

## Nanoparticle-Functionalized Antibiotics (nAbts)

### Available Types

The dosage of conventional antibiotics through nanoparticle-based delivery systems offers added advantages over the “free” antibiotics, such as reduced frequency and dose, sustained drug release contributing to increased antibacterial efficiency, and targeted intracellular drug delivery localized site inside bacteria. To date, antibiotics encapsulated as nanoengineered systems have been investigated as the delivery vehicle of a single or a combination of multiple antibiotics. These systems include (i) membrane-bound NPs (e.g., liposomes, dendrimers, micelles), (ii) polymeric NPs (PNPs), (iii) lipid polymer hybrid NPs (LPHNs), (iv) solid metallic NPs, (v) metal-oxide NPs, (vi) chitosan NPs (CSNPs), (vii) mesoporous NPs (viii) nanostructured particles manufactured from carbon-based materials (e.g., graphenes, carbon nanotubes), and other NPs such as nanocomposites (NC), nanosheets (NS), nanomesh, hydrocarbon (HC), and solid lipid NPs (SLNs), etc. Among all the NP types, membrane-bound micelles or liposomes, polymeric, and mesoporous systems are the most studied delivery systems.

**FIGURE 2:**
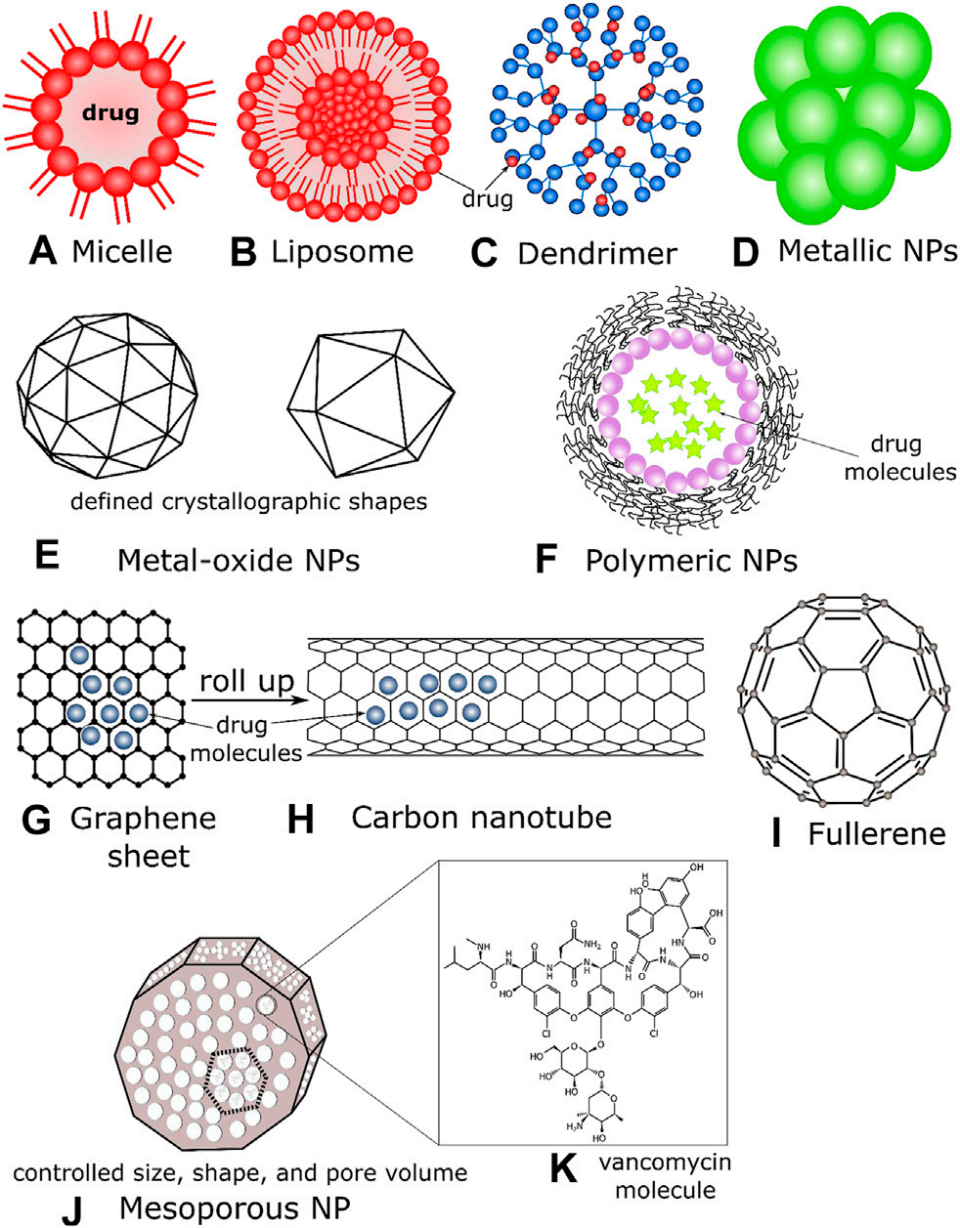
Schematics of **(A)** a micelle, where drug molecules assemble to the polar heads of the lipid, **(B)** a liposome, drugs can be functionalized/contained inside the lipid bilayer, **(C)** a dendrimer containing a core moiety and branched polymers (active terminal surfaces) where antibiotics can be sequestered, **(D)** metallic NPs, usually composed of inorganic solids, drugs can be incorporated on their outer surfaces, **(E)** metal oxide NPs have definite crystallographic architectures; their metallic and/or magnetic properties act as efficient drug carriers and penetrates the bacterial cell membranes effectively, **(F)** polymeric NPs encapsulating drug molecules, **(G)** a graphene sheet (2D) with drug molecules, **(H)** a carbon nanotube that can be formed by rolling graphene sheets, **(I)** a fullerene NP (C60) **(J)** a mesoporous NP, where the individual size, shape, and pore volume can be controlled depending on the antibiotic’s structures **(K)** the molecular structure of vancomycin antibiotics depicted to be in the pore and void volume of mesoporous NPs.

Among all the available existing antibiotics, vancomycin (van), a highly cross-linked branched glycopeptide antibiotic, is the most studied drug that has been enormously explored for its *in vivo* and *in vitro* activities in different bacterial cells and biofilms, with the synergy of nanostructured systems (Pichavant et al., 2016; Yeh et al., 2020). Although primarily used against the genus *Staphylococcus*, vancomycin is ineffective against Gram-negative organisms (Fernandes et al., 2017). However, the NPs structured synergy with van, the conjugates can be a potent bacterial eradicator against most Gram-negative microbes and some MDR bacteria (Pichavant et al., 2016; Murei et al., 2020; Gu et al., 2003; Chen et al., 2018; Fernandes et al., 2017). Gram-negative bacteria possess an external layer of cell membrane composed of lipopolysaccharide impenetrable by van. Nevertheless, the nano-transformation of van can overcome the intrinsic resistance of Gram-negative bacteria (Fernandes et al., 2017). Van inhibits external cell wall synthesis of Gram-positive bacteria without being released from the nanoconjugate systems and can be covalently bound onto biomaterials. Van efficiently binds with microbial cell wall structures, more favorably with the D-Ala-D-Ala terminal moieties of bacterial peptidoglycan, inhibiting cell growth (Wang et al., 2018a). This hydrophilic molecule can from multiple strong hydrogen bond interactions with various biomaterials. Therefore, vancomycin is a model antibiotic to study nano drug-delivery advances and reverse antibiotic resistance (Kell et al., 2008). In *Membrane-Bound* to *Other NPs* sections, we summarized the different types of antibiotics-nanoparticle conjugate systems, their associated chemistry, and antibacterial mechanisms. Figure 2 depicts common NP types functionalized with antibiotics to deliver drug molecules at bacterial target sites.

#### Membrane-Bound

Membrane-bound NPs are emerging solutions for drug delivery because they can interact directly with the bacterial host cell’s surface membranes. Additionally, membrane-bound NPs can encapsulate amphiphilic, hydrophobic, and hydrophilic drugs, but primarily lipophilic antibiotics (Lee and Thompson, 2017; Caminade, 2014). The lipid bilayer of liposomes can encapsulate lipophilic antibiotics at the desired concentration and deliver them to the infection site without losing their potency and exerting undue toxicity (Gonzalez Gomez and Hosseinidoust, 2020). Therefore, liposomal delivery reduces antibiotics’ footprint in the environment, a crucial factor that can limit the evolution of antibiotic resistance in bacterial mutant strains in the environment (Hocquet et al., 2016). Micelles are specialized liposomes with a lipid monolayer instead of a bilayer. Hydrophobic antibiotics can be functionalized inside a reverse micelle’s polar core or a normal micelle’s polar surface. The size of liposomes and micelles for drug delivery purposes typically falls in the range of 50–200 nm and 5–100 nm, respectively (Martins et al., 2016). A dendrimer’s size is even smaller (1–20 nm), which connects one central core surrounded by many layers of branched macromolecules with active terminal surface groups (Paleos et al., 2010). Dendrimers form many hydrophilic or hydrophobic holes that can simultaneously encapsulate single or multiple antibiotics, depending on the active terminal surface groups’ functional moieties. The hydrophobic interactions and self-aggregation of amphiphilic molecules into nanostructured vesicles can carry high drug loads when multivalent charges are present on the nanoparticle surface (Yadav et al., 2020). Lipid coated nAbts, which contain both the hydrophobic membranes and hydrophilic cores, can be functionalized to entrap amphiphilic antibiotics simultaneously. Table 1 shows three types of membrane-bound antibiotics-nanoparticle conjugate systems, their associated chemistry, and antibacterial mechanisms.

#### Metallic

Metallic NPs are popular in biomedical research fields due to their ease in synthesis and surface functionalization, plasmonic features, and spherical shape, allowing the development of nano-based drug design and delivery process and mechanism studies (Patra et al., 2018). Silver and gold are the most studied and widely used metals for nanoformulations (Mody et al., 2010). Metallic NPs form non-covalent hydrogen bonding with antibiotic molecules. This metal-molecule conjugates primarily destroy bacteria by disrupting the cell membrane’s physical barrier with a surface adhesion and subsequent passive diffusion mechanism (Chen and Bothun, 2014). Table 2 shows two major types of metallic NPs and their associated functions.

#### Metal-Oxides

Metal-oxides NPs (MONPs) possess unique properties such as durability, stable net negative surface charge, easy functionalization with various drug molecules, precise engineering to custom-built particle morphology, resistance to swelling, facile entrapment of both hydrophilic and hydrophobic antibiotics, etc. The oxygen vacancies and variable defect sites (Ruiz Puigdollers et al., 2017) can be constructed on-demand in MONPs, in which electrochemical and catalytic active sites are present. This on-demand modification allows for multiple oxidation states/polyvalency and crystallographic coordination and extensive functionalization of antibiotic molecules. Additionally, paramagnetic species (e.g., iron oxide NPs) are common in MONPs that can provide high entrapment efficiency of drug molecules due to Van der Waals interaction, dipole-dipole attraction, and hydrogen bonding. The increased surface energy and net negative multivalent surface charge in the crystal allow passive diffusion, which perturbs the cell membrane structures, resulting in oxidative stress (Esmaeili and Ghobadianpour 2016; Armijo et al., 2020). Table 3 includes three metal oxide types and their associated functions. Bare magnetic NPs are nontoxic to bacteria; therefore, they are considered not microbicidal. However, loading them with antibiotics (e.g., vancomycin) can transform them into a potent antibacterial agent (Kell et al., 2008). Table 3 shows three different MONPs and their associated functions.

**TABLE 3:** Different types of metal-oxides nAbts and their associated functions.

Metal-Oxides NPs (MO-NPs)		Size/shape	Conjugated antibiotic	Conjugate’s chemistry	Targeted bacteria	Target site	Mechanism of action	Reference
CeO_2_		5–20 nm, spherical	β-lactam (cefotaxime, imipenem, amoxicillin, clavulanate, ampicillin)	CeO_2_ NP or nanoceria possess defect sites on its surface and oxygen vacancies that can load high antibiotic molecules to be functionalized.	*Klebsiella pneumoniae* carbapenemase KPC (KP1/11), *Escherichia coli* HB101	Outer cell membrane	Increases the outer cell membrane permeability by passive diffusion of antibiotics; oxidative stress by Ce^3+^ or Ce^4+^ ions to the cell membrane.	Bellio et al. (2018)
Superparamagnetic NPs (Iron-oxide)	MnFe_2_O_4_	25 nm, spherical	Vancomycin (Van)	MnFe_2_O_4_ NPs were coated with chitosan (Ch) and PEGylated. Mn-O bond has multiple oxidation states, and Mn-O-Fe interacts as a metalloenzyme with the amide-amide hydrogen bonding of Van to form Van-PEG-Ch- MnFe_2_O_4_ NPs	*Staphylococcus aureus* (ATCC25923), *Staphylococcus epidermitis* (ATCC12228), *Bacillus subtilis* (ATCC1720), *Escherichia coli* (ATCC1399), *Pseudomonas aeruginosa* (ATCC1430), MRSA (ATCC33591)	Outer cell membrane	Intracellular proteins and Lactatedehydrogenase leakage from the cell membrane, oxygen-free radical generation, and disruption of mitochondrial functions. Slow, sustained release of Van-PEG-Ch-MnFe_2_O_4_ NPs require a lower concentration of Van to achieve the antibacterial activity, thus reduces the scope of antibiotic resistance development.	Esmaeili and Ghobadianpour (2016)
	Fe_3_O_4_	15–45 nm, oblong, rounded-rectangular	Tobramycin	Functional groups of alginate-coated Fe_3_O_4_ NPs conjugated with the primary amines (-NH2) group of tobramycin.	*Pseudomonas aeruginosa*	Outer layer of the biofilm	Unusually high negative surface charge of alginate coated Fe_3_O_4_ NPs undergo a diffusion process crossing the outer layer of biofilm, which is also negatively charged. Alginate (polymer) coating on iron-oxide NPs provides rapid bio-uptake by biofilm barrier and delivers tobramycin at the target site.	Armijo et al. (2020)

#### Carbon-Based

The literature on carbon-based nAbts is quite limited (Carver et al., 2020; Singh et al., 2019) as they are not being widely studied. However, they have enormous adsorption capacities suitable as delivery vehicle of pharmaceuticals. Graphene is the most common form of carbon-based NPs, rolling their sheet-like structures into cylindrical cages form carbon nanotubes (CNTs). Interestingly, the average size of single-walled CNTs or graphene is typically 200–500 nm. Although larger than a nanoparticle, such an increase in size confers them added advantages. For instance, bacterial efflux pumps are much larger than their membrane lipid monomers. When a CNT or a nanographene oxide (NGO) penetrates the cell membrane by physically breaking the barrier, they are trapped inside. The efflux pumps cannot eject the drug-carrying nAbts (Carver et al., 2020). Table 4 shows two major carbon-based nAbts and their associated functions.

**TABLE 4:** Different types of carbon NP based nAbts and their associated functions.

Carbon nanomaterial. Based NPs		Size/shape	Conjugated antibiotic	Conjugate’s chemistry	Targeted bacteria	Target site	Mechanism of action	Reference
Graphene oxide (C_140_H_42_O_20_)	Nanographene oxide (NGO)	500 nm, Rectangular shape	Tetracycline (TET)	Strong adsorptive attachment of NGO surface with TET by Van der Waals forces, π-π electron-donor-acceptor interactions, and cation-π bonding.	*Escherichia coli*	circumvent the efflux pump membrane proteins	NGO carries TET into the cytoplasm to bind with the ribosome. The size of NGO is larger than efflux pump membrane proteins. Thus TET is retained inside the cell, inhibiting bacterial growth.	Carver et al. (2020)
	Graphene oxide (GO)	200 nm, sheet type	Vancomycin (VAN)	Free -NH_2_ groups of VAN covalently bond with -COOH groups of GO. VAN molecules bind on the surface of GO.	Vancomycin-resistant *Staphylococcus aureus* (VRSA)	Cell membrane and cell wall	VAN-GO conjugate can deliver a very high amount of cationic VAN upon their release and interactions with negatively charged bacteria, and thus increased turgor pressure damages membrane by ROS generation. Inhibition of cell wall shrinkages the cell, followed by cell lysis.	Singh et al. (2019)
	Graphene oxide (GO)	600 nm, flakes	Linezolid (LZD)	In the absence of aminic groups, poor adsorption of LZD on the GO surfaces increases drug efficacy and enhances release potential.	*Mycobacterium tuberculosis*, Mtb H37Rv	Alveolar macrophages	LZD remains effective, being poorly adsorbed on GO surfaces, can be readily released inside the infection site. GO increases the preferential accumulation of LZD in the lungs and can block extracellular Mtb entry into the macrophages, which hereby diminishes infection. GO-LZD co-administration has increased anti-tuberculosis activity and may reduce the emergence of antibiotic resistance. Interestingly, GO-LZD has a two to three-fold surge in ROS generation than LZD alone.	De Maio et al. (2020)
Carbon nanotubes (CNT)	Single-walled carbon nanotubes (SWCNT)	500 nm, Cylindrical needle-type shape	Tetracycline (TET)	Strong adsorption of tetracycline’s NH_2_—bond with SWCNT	*Escherichia coli*	circumvent the efflux pump membrane proteins	Needle-shaped SWCNT can penetrate the bacterial barrier more efficiently and deliver TET into the cytoplasm to bind with ribosomes with greater efficacy. The size of SWCNT is larger than efflux pump membrane proteins; thus, TET is retained inside the cell, inhibiting bacterial growth.	Carver et al. (2020)

#### Chitosan

Chitosan (CS), a biopolymer, is the second most abundant naturally occurring linear amino-polysaccharide. CS-based nAbts are an excellent choice for antibiotics functionalization and delivery due to their enhanced biocompatibility, bio-uptake ability, biodegradability, and are non-toxic (Naskar et al., 2019). The high affinity of CS for cell membranes makes CS an effective targeting agent for bacterial drug delivery while entrapping antibiotic molecules. Chitosan is primarily positively charged because of the protonation on the primary amine groups. Covalent and electrostatic bonding is common for CS because it can form nanosystems with a high number and density of surface cations. CS-nAbts are spherical, with sizes ranging from 30–130 nm. CS loaded with penicillin or gentamicin can eradicate *Staphylococcus* and *E. coli* species. 2,6-Diamino chitosan (2, 6-DAC) has limited biocompatibility; however, with novobiocin and 2,6-DAC, its hydrophilicity significantly increases. This functionality results in high surface cations that reduce antibiotic resistance in many species, such as MRSA, *Staphylococcus aureus*, *Klebsiella pneumoniae, Acinetobacter Baumannii*, and *Pseudomonas aeruginosa* (Si et al., 2021). The net high positive surface charges of CS-nAbts promote enhanced surface binding combined with the bacterial cell membrane’s negative surface charge. Ultimately, the bacterial outer membrane lipid and peptidoglycan layers disassemble due to the contact killing mechanism. Table 5 shows three different CS-nAbts, their associated chemistry, and antibacterial mechanisms.

**TABLE 5:** Different types of chitosan-based nAbts and their associated functions.

Chitosan NPs	Size/shape	Conjugated antibiotic	Conjugate’s chemistry	Targeted bacteria	Target site	Mechanism of action	Reference
Poly (D-glucosamine) chitosan	124 ± 17 nm, spherical	Penicillin/streptomycin	Covalent bonding between chitosan and penicillin/streptomycin	*Staphylococcus epidermidis* (ATCC35984)	Cell wall and cell membrane	The electrostatic attraction of the polycationic nature (high surface charge density) of chitosan tightly adsorbs onto the bacterial cell wall’s anionic components. When synergized with CS nanocarrier, the slow-release kinetics of antibiotics ensures sustained drug delivery, disrupting the cell membrane, ultimately rapid cell death occurs.	Piras et al. (2015)
2, 6—diamino chitosan	80 nm, spherical	Novobiocin	The 6-position hydroxyl group of chitosan binds with novobiocin.	*Listeria monocytogenes*, *MRSA USA300*, *Staphylococcus aureus* (ATCC29213), *Klebsiella pneumoniae* (BAA2784)*, Acinetobacter Baumannii* (ATCC17978, and BAA-2803), *Pseudomonas aeruginosa* (BAA2797), *Pseudomonas aeruginosa* PA01	Cell wall	The synergy of chitosan with novobiocin brings additional amino groups in the conjugate. This synergy increases the hydrophilicity of chitosan and enhances the proton sponge effect (higher cationic charge), resulting in enhanced antibacterial efficacy.	Si et al. (2021)
Chitosan/Fe_3_O_4_/Poly (ethylene glycol) PEG	30 nm, spherical	Gentamicin (Gent)	CS-loaded Fe_3_O_4_ NPs are combined with Gent by strong electrostatic interactions. The dicarboxylic acid groups of PEG bind CS- Fe_3_O_4_ NPs loaded Gent with PEG.	*Staphylococcus aureus*, *Escherichia coli*	Cell membrane	Gent release from Fe_3_O_4_-PEG NP is pH-dependent and greatly enhanced at low pH (5.5–6.5). This dependency suggests a high diffusion of Gent into the surrounding environment when pH is acidic and subsequent destruction of the cell membrane occurs. The CS and PEG groups of NPs induce positive surface charges to the bacterial membrane's negative surface charges, promoting a contact killing mechanism by the nanocomposites themselves.	Wang et al., (2018b)

#### Polymeric

Most polymeric NPs are by themselves antimicrobials (Richards et al., 2018). PLGA—poly (D, L-lactide-co-glycolide) are the copolymers of glycolic and lactic acid (Essa et al., 2020). They are biodegradable and biocompatible NPs with target-specific delivery potentials inside cells or organs (Soppimath et al., 2001; Gagliardi et al., 2021). Modification of these NP’s surfaces can offer stimuli-sensitive responses or target specific features―which may modulate their biodistribution and site-specific target and concomitant uptake pathways in bacteria. Preferential accumulation and cellular internalization are the two crucial properties of nanoconjugate systems that confer added advantages on top of the antibiotic itself. Enhanced permeability and retention (EPR) effects of PLGA-enabled antibiotics mainly affect multiple biological factors of a bacterial cell, target a site of infection or wound (Liu Y. et al., 2020), and have been shown to decrease antibiotic resistance burden. PLGA polymeric NPs, in the 100–300 nm size range, can traffic through endothelial or epithelial mammalian infected cells and efficiently perform intracellular sustained drug delivery (Panyam et al., 2002). Infectious agents that cause persistent infections and chronic diseases such as *Chlamydia trachomatis* can be successfully reduced in clinical studies by PLGA nAbts with sustained release and enhanced drug delivery to the chlamydial inclusion complexes. (Toti et al., 2011). Table 6 shows five different polymeric-nAbts, their associated chemistry, and antibacterial mechanisms.

**TABLE 6:** Different types of polymer-based nAbts and their associated functions.

Polymeric NPs	Size/shape	Conjugated antibiotic	Conjugate’s chemistry	Targeted bacteria	Target site	Mechanism of action	Reference
PLGA	260 nm, spherical	Azithromycin, Rifampin	Each PLGA NP system can load 25% rifampicin or azithromycin, by weight, through surface conjugation.	*Chlamydia trachomatis*	*C. trachomatis* infected live cells.	Dual mechanisms of action: firstly, enhanced efficacy of combined azithromycin and rifampin therapy reduces chlamydia burden. Secondly, sustained drug release at the acute and persistent inclusion sites. Due to different drug release kinetics, only 25% of azithromycin and 12% rifampin can be delivered in the first three days. This lessened drug load at the infection site may subvert antibiotic resistance.	Toti et al. (2011)
PLGA	50–400 nm, spherical	Rifampicin	Each PLGA NP conjugate can encapsulate 10–30% Rifampicin, by weight, through surface functionalization.	*Mycobacterium bovis* BCG	Macrophage and phagolysosome	PLGA-rifampicin conjugates have a unique mechanism of internalization and intracellular trafficking. Once internalized in the macrophages, the conjugates reside in the phagolysosomes for 6–7 days. This sustained slow release of rifampicin kills BCG mycobacterium in the infected cells. Such a lower drug dose is less susceptible to antibiotic resistance development.	Kalluru et al. (2013)
Surface charge-switching PLGA-PLH-PEG (D, L-lactic-co-glycolic acid)-b-poly (L-histidine)-b-poly (ethylene glycol)	196 ± 7.8 nm, spherical	Vancomycin (Van)	Van is encapsulated onto the NP surface by solvent evaporation/double emulsion method.	*Escherichia coli* (ATCC11229), *Staphylococcus aureus* (ATCC25923)	Cell wall	Under basic conditions, negatively charged NPs do not interact with non-targeted sites. Only under acidic conditions (pH 5.5–6.0), the PLGA-PLH-PEG NP’s PLH (imidazole group) part exponentially gains positive surface charges. These anions actively attach the targeted sites (negatively charged bacterial cell wall elements) in a strong multivalent electrostatic binding. Eventually, strong antibacterial effects occur by the controlled release of vancomycin.	Radovic-Moreno et al. (2012)

#### Mesoporous

Mesoporous NPs are amorphous solids with various inner pore diameters and void volumes. An ordered mesoporous structure has uniform pore diameters and void volumes. Mesoporous silica is an excellent example of a nanoparticle that sequesters antibiotics inside porous materials (Croissant et al., 2018; Selvarajan et al., 2020). Therefore, mesoporous silica exhibits high drug loading capacities. Functional organics can be incorporated in the inner walls (pores) of mesoporous silica to conjugate molecules of interest (Doadrio et al., 2006). For example, macrolide antibiotic molecules that treat acute and chronic infections contain long alkyl chains (Dinos and George, 2017). Controlled delivery of these long-chain alkyl molecules is achieved by functionalizing them onto mesoporous silica. This functionalization results in a diminished degradation rate and enhanced hydrophobicity of the resulting conjugate (Doadrio et al., 2006). An outstanding feature of mesoporous nAbts that distinguishes them from other types is their potential for differential drug release. The high number of inner cavities creates electron-deficient or electron-rich areas. Depending on this property, oppositely charged antibiotics can be trapped in those cavities, and their concentrations can be adjusted by controlling the size and shape of the void spaces. Although electrostatic interactions between the particle surface and antibiotics dominate the mesopores, hydrogen bonds form with the silica surface (Si-O bonds) and drug molecules as well. By altering the mesopores' morphology, release kinetics and diffusion dynamics can be controlled with the sequestered antibiotics. Table 7 shows seven different mesoporous nAbts, their associated chemistry, and antibacterial mechanisms.

**TABLE 7:** Different types of mesoporous nAbts and their associated functions.

Mesoporous NPs	Size/shape	Conjugated antibiotic	Conjugate’s chemistry	Targeted bacteria	Target site	Mechanism of action	Reference
Silica	72.4 ± 8.2 nm, quasi-spherical	Polymyxin B, Vancomycin	Positively charged polymyxin B and vancomycin adsorbs in the cylindrical holes of negatively charged bare-MSNs and carboxyl modified MSNs *via* electrostatic interactions. Pore size and available surface area inside the holes determine the concentration of antibiotics to be absorbed.	*Staphylococcus Aureus* (DSM 20231) *Escherichia Coli* (K12 DSM498–0714-001) *Pseudomonas aeruginosa* (PAO1 DSM 19880) *Klebsiella oxytoca* (DSM 5175) *Acinetobacter Baumannii* (DSM 30006)	Outer cell membrane	Synergistic activity of polymyxin B and vancomycin increase the antibiotic potency both on gram-positive and gram-negative bacteria. Polymyxin B interacts with Gram-negative bacteria’s outer membrane; Van disrupts peptidoglycan synthesis. The lower release rate of carboxyl loaded MSNs (containing higher net negative charge) enhances the antibacterial efficacy by the increased local concentration of immobilized antibiotics.	Gounani et al. (2019)
Silica core-shell	277 ± 12 nm, spherical	Gentamicin sulfate and sodium rifamycin	Positively charged gentamicin adhesion on the silica core NPs (first antibiotic loading). Three OH groups of gentamicin favor this interaction on the silica surface by hydrogen bond formation. The shell functionalization with thiol (R-SH) group favors negatively-charged rifamycin sorption on the outer surface like a shell layer (second antibiotic loading).	*Staphylococcus aureus* (ATCC29213), *Pseudomonas aeruginosa* (ATCC27853)	Cell membrane	Two oppositely charged antibiotics can be delivered using the silica core-shell NP, with different release kinetics of two drug molecules. Rifamycin is rapidly desorbed; on the other hand, gentamicin requires a longer time to release and follow a slow diffusion pattern. For Gram-positives, core-shell NP can deliver dual antibiotics effectively and show 1.5 × more potency than a single antibiotic.	Mebert et al. (2016)
Mesoporous silica NPs (MSNs) with Cu (II) and Ni (II) complexes	<100 nm, spherical	Gentamicin	NH_2_ and COO^-^ groups of gentamicin interact with the free coordination sites of Cu (II) and Ni (II) complexes supported on MSNs nanochannels, resulting in gentamicin’s high adsorption.	*Staphylococcus aureus* (ATCC6538), *Bacillus subtilis* (ATCC6633), *Pseudomonas aeruginosa* (ATCC9027), *Escherichia coli* (ATCC25922)	Cell membrane	Tiny, porous structures of metal-MSNs complexes enable high adsorption of gentamicin, and as a drug carrier delivers increased gentamicin in the cell membrane. Also, they facilitate enzyme immobilization.	Tahmasbi et al. (2018)
Ordered mesoporous silica NPs (OMSNs)	100 nm, Non-spherical (oblate)	Isoniazid (INH)	INH encapsulates into the hollow oblate structures of OMSNs, and functionalization with trehalose sugar provides specific targeting ability to mycobacteria	*Mycobacterium smegmatis* mc^2^ 651	Cell wall	Enhanced interactions of INH loaded OMSNs with the bacterial cell. The OMSNs have anisotropic morphology, low density, high surface-to-volume ratio, and large hollow interior capacity that enhance cell binding efficiency (adhere to bacterial cell surfaces), cellular uptake kinetics, high drug-encapsulation capacities, sustained drug release, and increased interactions of particles with bacteria.	Hao et al. (2015)
Carbon	Pore size 5.8 and 13.9 nm, wall thickness 25 and 45 nm, spherical	Vancomycin	Intrinsic hydrophobicity of mesoporous hollow carbon (MHC) allows higher vancomycin loading capacity in the porous nanospheres. By adjusting the pore size and wall thickness of MHC, vancomycin adsorption and release rate can be controlled.	*Escherichia. coli*, *Staphylococcus epidermidis*	Cell membrane	The combination of specific pore size and the wall thickness of MHC nanospheres contain higher vancomycin loading by physisorption and sustained drug release capacity over a long time to inhibit bacterial peptidoglycan synthesis. Adhesion of hydrophobic MHC nanospheres disrupts the cell membrane, followed by inserting vancomycin inside the cell.	Nor et al. (2016)
Titania-silica composites	Fiber or rope-like structures	Oxytetracycline (OTC)	Titania ions in silica wall surface create strong donor-acceptor bonds with OTC molecules.	*Staphylococcus aureus* (ATCC25923), *Escherichia coli* (ATCC25922) *Pseudomonas aeruginosa* (ATCC27853)	Cell membrane	Mesoporous crystalline titania on the silica surface allows slower OTC release for a sustained period inducing a burst effect.	Georgescu et al. (2017)
Silica NPs	50–80 nm, spherical	Tetracycline (TC)	Silica-tetracycline composites (SiO_2_-TC) forms by the silanol (Si-O-H) group’s interaction with TC molecules inside silica pores.	TC/Amp resistant*Escherichia coli*, TC resistant *Escherichia coli*	Cell membrane lipopolysaccharides	TC NPs interact with lipopolysaccharides (create hydrogen bonds between saccharides and hydroxyl groups) and destabilize the silica surface’s peptidoglycan layer. TC stops protein synthesis by binding with the 30S ribosome subunit.	Capeletti et al. (2014)

#### Other NPs

Several other NPs types are primarily designed and subsequently developed to overcome some of the challenges of functionalized drugs with NPs discussed above. These challenges include limited solubility/dispersibility, reducing the concentrations of non-specific antibiotic release, promoting co-delivery of multiple antibiotics, avoiding toxicological consequences, improving colloidal stability, ability to bind with a specific ligand or surface-active group, biodistribution, improving the localized delivery of functional compounds, enhancing biodegradability and biocompatibility, etc. For instance, nanosheets are composed of a single or multiple layers of 2-D array of atoms or molecules (a graphene sheet or montmorillonite). Their external surfaces are excellent platforms for multivalent ion complexation or chelation. Ion dissolution from the cation-exchange sites heavily overloads the bacterial cells causing a ruptured or disintegrated membrane (Chahardahmasoumi et al., 2019). Table 8 includes six different types of NPs used to functionalize various drugs and their associated functions.

**TABLE 8:** Other types of nAbts and their associated functions.

Other types of NPs	NPs	Size/shape	Conjugated antibiotic	Conjugate’s chemistry	Targeted bacteria	Target site	Mechanism of action	Reference
Nanocomposite (NC)	Ag	Ag 15 ± 5 nm, quasi-spherical	Tetracycline (TC)	Ag chemically binds with amide group of TC	*Escherichia coli* K12 ATCC 29425 (S), *Escherichia coli* ST648 (R), *Staphylococcus aureus* ATCC 25923 (S)	Outer cell membrane	Ag cytotoxicity in bacterial cells with TC as protein synthesis inhibition brings synergistic dual antimicrobial mechanism.	Djafari et al. (2016)
Nanosheets	Montmorillonite	40–120 nm, sheet	Tetracycline (TC)	At low pH, tetracycline absorbs on the external surface by complexation/chelation with Al^3+^, Fe^2+^/Fe^3+,^ Si^4+^, Mg^2+^ ions. Furthermore, diffuse into interlayer spaces by the cation exchange mechanism.	*Escherichia coli*, *Staphylococcus aureus*	Outer cell membrane	Dissolved metal ions release and multivalent cations acquisition overload crossing outer membrane channel.	Chahardahmasoumi et al. (2019); Hohle et al. (2011)
Nanomesh	Gold NPs	5 nm, spherical	Vancomycin (Van), Colistin/Polymyxin E	Using an electrospinning method, the drug’s charge and the functionalization of charged particles within a nanofiber/nanomesh system enable the drug positioning within a mesh.	*Escherichia coli*	Outer cell membrane	The opposite charge of Gold NPs to the drugs can increase the release from the Nanomesh. Increased burst release of dual drugs diffuses from the mesh in the first few hours and subsequently has different sustained release rates.	Fuller et al. (2019)
Hydrocarbon	Norbornene (Nb) NPs	50 nm, spherical	Vancomycin (Van)	NPs are synthesized by ring-opening metathesis copolymerization (ROMP) of Nb-Van (polyethylene oxide). Amide bond of Van functionalized with Nb by a covalent link on a titanium surface.	Methicillin-resistant *Staphylococcus aureus* (MRSA BCB8)	Cell wall	The multivalent polymer of the Van-Nb NP system can increase the specific surface and deliver 1.2 × 10^6^ van molecules/NP, such high Van density at a specific site enhances the antibacterial activity.	Pichavant et al. (2016)
Solid Lipid (SLNs)	Lipid NPs (L-NPs), Phospholipid NPs (PL NPs)	125–175 nm, spherical	Penicillin-G (PenG), levofloxacin (Levo)	Two stearoyl chains of alkane-based lipids are connected with the antibiotic’s extended hydroxyl groups. Diacetyl phosphate groups of phospholipids are conjoined with R-O groups of antibiotics.	Methicillin sensitive*Staphylococcus aureus*	Cell membrane	PenG-PL NPs can load a high amount of PenG with a rapid release rate. PL part attaches PenG to the cell membrane, thus achieve greater internalization inside the cell, killing intracellular bacteria.	Zhang et al. (2019)

### Future Potentials of Nanoparticle Functionalized Antibiotic Systems

Nanoengineered systems such as carbon quantum nanodots (CQDs), multiwalled carbon nanotubes (MWCNTs), fullerene particles (C_60_), nanoemulsions, and various polymer-based NPs have not been extensively studied for their capabilities for antibiotic synergy and delivery in bacteria. However, they hold immense potentials for drug functionalization, site-specific delivery inside bacterial cells, and designed bacterial killing. For example, zwitterionic carbon quantum dots can induce bacterial apoptosis and programmed cell death (Bing et al., 2016). Hydrophobic surfaces of cylindrical-shaped single and MWCNTs nanotubes and fullerenes can bind successfully with bacterial lipid bilayers and disrupt the membrane integrity leading to cell death (Azizi-Lalabadi et al., 2020). Most MWNCTs need modification to enhance their antibacterial functions. For instance, silver NPs functionalized with a dendrimer modified MWNCTs bring significant broad-spectrum antibacterial functionalities (Yuan et al., 2008). Table 9 summarizes the existing and future potentials of antibiotics-nanoparticle conjugate systems, their associated properties, and antibacterial mechanisms.

**TABLE 9:** Future potentials of nanoparticle-antibiotics synergy.

Nanoparticle	Antibiotics	Properties	Antibacterial mechanisms	References
Fullerene (C_60_)	Vancomycin	1. Unique carbon cage structures, size, hydrophobicity, electronic configurations, and three-dimensionality	1. Disruption of membrane integrity in bacteria	Bakry et al. (2007); Tang et al. (2007); Prylutskyy et al. (2014)
		2. Production of high quantum yield singlet oxygen	2. Fullerene interacts with the hydrophobic cavity of enzymes and thus inhibits enzyme activity	
		3. Can cleave DNA due to the electron transfer from excited state fullerene to DNA base	3. Induce oxidative stress	
		4. For hybrid nanostructures, fullerene provides high encapsulation efficiency with lipidic NPs	4. Perturb energy metabolism	
			5. Interact with cytochrome P450S, cysteine, and serine proteases	
			6. Cationic fullerenes react with negatively charged bacterial surfaces and the potential to disintegrate cell membranes by redox damage or mechanical breakage of the lipid bilayer	
Carbon quantum dots (CDQs)	Ciprofloxacin hydrochloride	1. High ciprofloxacin loading capacity	1. Controlled release of Ciprofloxacin at a slower rate from the surface of CDQs	Thakur et al. (2014)
		2. Avoid non-specific deposition, only reach the site of infection	2. Deliver high concentration of antibiotic	
		3. Can be used as a molecular-tag to locate the infection site in a host	3. Various functional groups of CDQs inhibit cellular proliferation	
			4. ROS generation from the charge-separated CDQs species	
Polymer	Penicillin, tetracycline, sulfonamide, fluoroquinolones	1. Multifunctionality, good biocompatibility, and stable drug delivery both at in-vitro and in-vivo conditions	1. As opposed to free antibiotics, polymeric NPs functionalized with antibiotics can overcome tissue barriers and improved penetration through cell wall and membranes	Xiong et al. (2014)
		2. Improved biodistribution and pharmacokinetics of antibiotics	2. The synergy of polymeric NPs and antibiotics provide slow, sustained release of drug molecules at inaccessible specific site overcoming thick tissue layer	
		3. Provides environmental deactivation		
		4. Due to being bioactive in nature, dose and frequency can be reduced		
Multiwall carbon nanotubes (MWCNTs)	Vancomycin	1. MWCNT’s carboxyl group and Van’s amide group form a robust antibacterial conjugate	1. Can effectively breakdown various human gut microbe’s membranes	Chen et al. (2013); Liu et al. (2017)
		2. This powerful agent can kill both Gram-positive and Gram-negative bacteria	2. Rupture the DNA and RNA components, followed by the destruction of cell membranes	
			3. Inhibit the biosynthesis of cell wall peptidoglycan and may reduce RNA synthesis	
Nanoemulsions	Erythromycin	1. Overcome poor drug solubility	1. Increased drug entrapment and loading efficiency enable high concentrated localized drug delivery	Sutradhar and Amin. (2013); Tran et al. (2017)
		2. long term activity	2. Better drug absorption, penetration, and accurate dosing at target specific site ensure appropriate drug concentration, thereby reduces the chances of antibiotic resistance development	
		3. Target specific		
		4. High retention time		
		5. Require low dose		
		6. Erythromycin stability improves under acidic condition		
		7. Enhancement of bioavailability		
		8. Better absorption inside cellular systems		

## Future Research Perspectives

Antibacterial NPs target multiple biomolecules; therefore, nAbts will be an important intervention in halting the evolution of multi-drug resistant organisms (MDROs) (Slavin et al., 2017). NPs retain their morphological properties when combined with antibiotics (Mu et al., 2016). Hence, it is urgent to test nAbts against resistant strains. The interaction of specific NPs with repurposed therapeutics may allocate new compounds for treating MDROs. Extensive investigation on the potential of nAbts is crucial to circumvent antibiotic resistance from diverse microbial agents to prevent future pandemics.

The Trojan horse strategy, unique physicochemical features, and antibacterial mechanisms arising from NPs can be employed to treat infections (Abed and Couvreur, 2014) or even biofilms (Prasad and Gupta, 2020). Although most nanoconjugates exhibit microbicidal/antipathogenic/microbiostatic properties (discussed in *Nanoparticle-Functionalized Antibiotics* section), they seldom reach clinical studies or are made available to clinicians. Consequently, our limited resources cannot fight the resurgence of multidrug resistance if we only rely on molecular pharmaceuticals. Decades of studies on nanomedicine approaches (Kinnear et al., 2017) have improved our knowledge on bacterial burdens and infectious diseases (Yang et al., 2019), at least in murine models (Liang et al., 2016). But to date, and to our knowledge, no nanomedicine-based antimicrobial therapy has been accepted for treating resistant microbial infections in humans.

Some of the severe and urgent threats mentioned by the Centers for Disease Control and Prevention (CDC) include vancomycin-resistant *Enterococcus* (VRE), vancomycin-resistant *Staphylococcus aureus* (VRSA), methicillin-resistant *Staphylococcus aureus* (MRSA), multi-drug resistant *Pseudomonas aeruginosa*, and extended-spectrum β-lactamases (ESBL) producing Enterobacteriaceae (CDC, 2021). Drug-resistant species occasionally hide inside human cells and remain unrecognized by the immune system. One study reported that cerium oxide NPs can penetrate the membranes of affected cells (infected macrophages) and directly kill MRSA (Matter et al., 2021). They attributed the mechanism to cerium oxide NPs dissolving and eliminating MRSA from their cellular hideouts (Matter et al., 2021). But selective targeting is critical since most NPs kill bacteria randomly, including beneficial bacteria that can produce NPs as well. For instance, *Staphylococcus aureus* (SA) can be programmed to yield proteinaceous NPs. Selenium NPs are lethal to SA and *E. coli* (Huang et al., 2019; Geoffrion et al., 2020). NPs generated from SA or *E. coli* can be engineered as a nanometric Trojan horse to kill MRSA, VRSA, VRE, and drug-resistant *E. coli*.

GO has been successfully coupled with Linezolid to treat *Mycobacterium tuberculosis* (De Maio et al., 2020), also known as multi-drug-resistant TB or extensively drug-resistant TB (XDR-TB). Metronidazole could be combined with GO and biomimetic PLGA or membrane-bound NPs to treat *Clostridiodes* (clostridium) *difficile* infections. Ampicillin or penicillin is used to treat pneumococcal infections. β-lactams (e.g., penicillin or cephalosporin) are the first line of defense against *Pseudomonas aeruginosa* (Bassetti et al., 2018). Nano-penicillin (Yariv et al., 2015) or nano-ampicillin can be introduced to fight drug-resistant *Streptococcus pneumoniae*. Combinatorial beta-lactams (Siriyong et al., 2019) synthesized in pure-nano form as explained in (Yariv et al., 2015) may be applied to address multi-drug resistant *Pseudomonas aeruginosa*.

As described in this review, antibiotics encapsulated into nanocarriers bring numerous benefits, yet disadvantages exist. First, most materials used for developing nanosystems may be toxic (Tao, 2018) for clinical applications (Vimbela et al., 2017), except for a few bioinspired and biomimetic materials (Yang et al., 2019). Second, non-biodegradable inorganic and magnetic NPs may exhibit undesirable properties in humans, and their excretion from the body is a safety concern. Third, even though nAbts are commercially cheap, versatile, and exhibit a long shelf life, translating their use from laboratory to clinical applications is challenging.

Fourth, NPs synthesized from the pure molecular antibiotics by various pharmaceutical or engineering approaches, their nanoparticulate components and structural features are still poorly characterized. Studies are needed on crystal structure detail, biodistribution, elimination, and co-administration of specific antibiotics because these parameters are essential for understating nAbts *in vivo* behavior, fate, and efficacy. Establishing the morphology-property association will be advantageous for optimizing and simplifying the commercial preparation of existing pure nAbts. This study can accelerate the design and development of other nanoplatforms such as nanoaerosol- or nanopowder-based antimicrobials.

Fifth, most studies on pure nanodrugs have been implemented in cancer research (Kasai et al., 2012; Fan et al., 2018). Only one study refers to theranostic applications of pure ciprofloxacin nanodrug in eradicating drug-resistant *Escherichia coli* (Xie et al., 2017). It is worth noting that most nanoparticle-functionalized antibiotics will leave an NP footprint; therefore, eliminating them from the body is not guaranteed after treatment. *In vivo* pharmacokinetic studies should be conducted to assess the biodistribution, biodegradation, site-specific delivery, cellular internalization, co-administration, and elimination of pure nAbts. We predict that pure nAbts’ pharmacokinetics and pharmacodynamics should be similar to their molecular analog, unlike nanoconjugate systems.

Sixth, studies on the antibacterial properties of fullerenes, CDQs, MWCNTs, and nanoemulsions are still in their infancy. Although their antibacterial properties and nano-enabled functions are known, the extent to which they can slow down antibiotic resistance is unknown. Consequently, the effectiveness and long-term safety issues of these newly introduced nano-inventions need to be confirmed by comprehensive laboratory and clinical scale anti-infective research. In this aspect, emerging technologies such as high-resolution single-particle scanning flow cytometry (Goddard et al., 2006), nanoproteomics (Tiambeng et al., 2020), and nano secondary ion mass spectrometry imaging (Lovrić et al., 2017; Gyngard and Steinhauser, 2019) can be used.

Finally, it is urgent to expand further the morphological functions, novel property identification, and different types of old and newly discovered antibiotics in combination with various NPs. Biological and life sciences are advancing with the revolution of nanotechnology as a Frontier. Data mining, deep learning, and artificial intelligence technologies can be integrated to discover more antibiotic molecules (Stokes et al., 2020; Das et al., 2021) and their potential synergy with NPs to enrich the existing arsenal of antimicrobials.

## Concluding Remarks

Since the discovery of penicillin, many antibiotics have effectively controlled the transmission of many bacterial infections. However, decades of extensive use and misuse have rendered many antibiotics ineffective in preventing and halting infections, leading to a surge of resistant bacterial strains. The global emergence and spread of antibiotic resistance have become a preeminent public health challenge, combined with the shrinking pipeline of effective antibiotics to combat them. These concerns highlight the urgent need for developing alternative approaches. Developing new therapeutics to address multiple bacterial resistance is hindered by the resource- and time-intensive process. Additionally, research and development on new molecular scaffolds are lagging because the economic incentives for pharmaceuticals companies may not justify the long time and resources needed to develop, screen, and test new antibiotics.

nAbts strategy addresses these concerns by leveraging existing and extensively studied antibiotics. It utilizes existing antibiotics as a strategy but delivers them either coupled to NPs or reengineering existing antibiotics in their pure form as NPs. This strategy hinges on the Trojan horse effect to protect or encapsulate these antibiotics. These NP-based antibiotics can bypass bacterial cell membrane barriers and reach specific sites with a higher level of specificity and stability than “free” antibiotic molecules because of the intrinsic abilities of NPs. The unique size-, shape-, and composition-related properties of NPs can pose multiple simultaneous challenges to bacteria to prevent them from developing resistance. The interaction of nanoscale antibiotics with bacterial intracellular components is of central importance for their applications such as antibiotic delivery, drug carriers, cell membrane penetration to reach target sites, protein synthesis disruption, to name a few. Insights from this review on a potential strategy to circumvent antibacterial mechanisms when exposed to nanoengineered systems will provide an understanding of bacteria-antibiotics interactions. Growing lines of evidence suggest that nano-antibiotics appear as a promising alternative strategy for overcoming antibacterial resistance and treatments in clinical infections.
